# Supplementary material for: Attenuating Effects of Dieckol on Hypertensive Nephropathy in Spontaneously Hypertensive Rats
Source: Int J Mol Sci. 2021 Apr 19;22(8):4230. doi: 10.3390/ijms22084230 (PMC8073021; doi:10.3390/ijms22084230)
Supplement: Supplementary file 1 [file ijms-22-04230-s001.zip › ijms-1188331-supplementary.pdf]

Supplementary tables

Table S1. List of primer for quantitative polymerase chain reaction

Gene		Primer
Tgfβ	Forward	5'– GAGCCCTGGATACCAACTACTG –3'
	Reverse	5'– AACCCAGGTCCTTCCTAAAGTC –3'
Smad2	Forward	5'– GAACTCGGAGAGGTTCTGCTTA –3'
	Reverse	5'– CTCCCCTTCCTATATGCCTTCT –3'
Smad3	Forward	5'– GGGGCTCTGTACATACCTTGAG –3'
	Reverse	5'– AGAAACACTGGCACTCTGACAA –3'
AT1R	Forward	5'– TAGCCAAAGGAAGAGTCAGGAG –3'
	Reverse	5'– GGAACATAGCAAAGGGAGACTG –3'
Snail2	Forward	5'– GGCCTTTCTCCTCTTACTGGAT –3'
	Reverse	5'– TGTGATCCTTGGATGAAGTGTC –3'
Actb	Forward	5' – ACAAAGCTGTTCAGTGTCTCCA – 3'
	Reverse	5' – CTCCGTTTCCAGAATACACACA – 3'

sTable 2. List of Antibodies for Western blotting,

Antibody name (clone)	Company	Cat. No.	Antibody dilution
β-actin (C4)	Santa cruz Biotechnology	sc-47778	1:500
TGF-β (9016)	Abcam	Ab64715	1:500
pSMAD2/3 (D27F4)	Cell signaling	8828	1:1,000
SMAD2/3 (D7G7)	Cell signaling	8685	1:1,000
AGTR1	Invitrogen	PA5-20812	1:1,000

Supplementary figure

sFigure 1. Comparative analysis of ECE and DK administration on the reduction of expression of E-cadherin in the kidney of SHRs

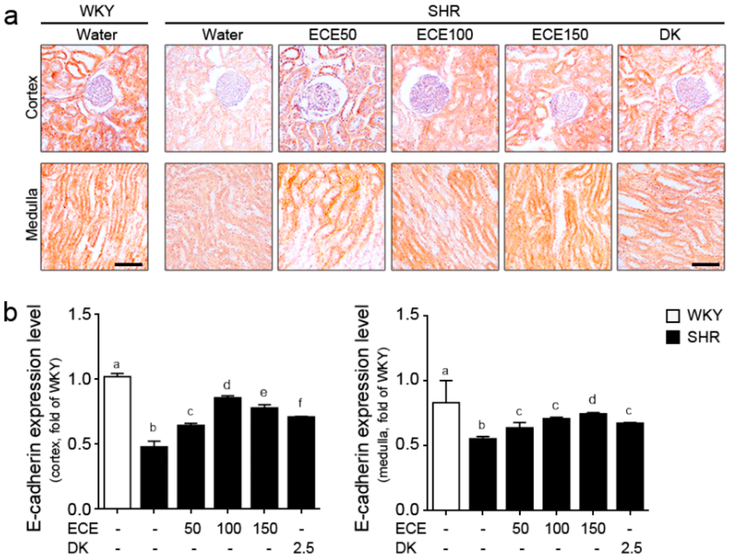

(a) E–cadherin protein expression level in cortex area, and in medulla of kidney were measured by immunohistochemistry and (b) the protein levels were quantified by Image J software. Scale bar = 50 μm. Three doses of ECE (50 mg/kg/day, 100 mg/kg/day and 150 mg/kg/day) was oral administrated for 4 weeks and 2.5 mg/kg/day of DK also oral administrated for 4 weeks. Means denoted by a different letter indicate significant differences between groups ($p < 0.05$). ECE, Ecklonia cava extract; DK, dieckol.
